# Core–shell polymeric nanoparticles co-loaded with photosensitizer and organic dye for photodynamic therapy guided by fluorescence imaging in near and short-wave infrared spectral regions

**DOI:** 10.1186/s12951-020-0572-1

**Published:** 2020-01-23

**Authors:** O. M. Chepurna, A. Yakovliev, R. Ziniuk, O. A. Nikolaeva, S. M. Levchenko, H. Xu, M. Y. Losytskyy, J. L. Bricks, Yu. L. Slominskii, L. O. Vretik, J. Qu, T. Y. Ohulchanskyy

**Affiliations:** 1 Key Laboratory of Optoelectronic Devices and Systems of Ministry of Education and Guangdong Province, College of Physics and Optoelectronic Engineering, Shenzhen University, Shenzhen, 518060 P. R. China; 2 Taras Shevchenko National University of Kyiv, Kyiv, 01601 Ukraine; 3 Institute of Organic Chemistry, National Academy of Sciences of Ukraine, Kyiv, 02094 Ukraine

**Keywords:** Polymeric nanoparticles, Short wave infrared fluorescence bioimaging, Photodynamic therapy, Electronic excitation energy transfer, Poly-*N*-isopropylacrylamide

## Abstract

**Background:**

Biodistribution of photosensitizer (PS) in photodynamic therapy (PDT) can be assessed by fluorescence imaging that visualizes the accumulation of PS in malignant tissue prior to PDT. At the same time, excitation of the PS during an assessment of its biodistribution results in premature photobleaching and can cause toxicity to healthy tissues. Combination of PS with a separate fluorescent moiety, which can be excited apart from PS activation, provides a possibility for fluorescence imaging (FI) guided delivery of PS to cancer site, followed by PDT.

**Results:**

In this work, we report nanoformulations (NFs) of core–shell polymeric nanoparticles (NPs) co-loaded with PS [2-(1-hexyloxyethyl)-2-devinyl pyropheophorbide-a, HPPH] and near infrared fluorescent organic dyes (NIRFDs) that can be excited in the first or second near-infrared windows of tissue optical transparency (NIR-I, ~ 700–950 nm and NIR-II, ~ 1000–1350 nm), where HPPH does not absorb and emit. After addition to nanoparticle suspensions, PS and NIRFDs are entrapped by the nanoparticle shell of co-polymer of *N*-isopropylacrylamide and acrylamide [poly(NIPAM-*co*-AA)], while do not bind with the polystyrene (polySt) core alone. Loading of the NIRFD and PS to the NPs shell precludes aggregation of these hydrophobic molecules in water, preventing fluorescence quenching and reduction of singlet oxygen generation. Moreover, shift of the absorption of NIRFD to longer wavelengths was found to strongly reduce an efficiency of the electronic excitation energy transfer between PS and NIRFD, increasing the efficacy of PDT with PS-NIRFD combination. As a result, use of the NFs of PS and NIR-II NIRFD enables fluorescence imaging guided PDT, as it was shown by confocal microscopy and PDT of the cancer cells in vitro. In vivo studies with subcutaneously tumored mice demonstrated a possibility to image biodistribution of tumor targeted NFs both using HPPH fluorescence with conventional imaging camera sensitive in visible and NIR-I ranges (~ 400–750 nm) and imaging camera for short-wave infrared (SWIR) region (~ 1000–1700 nm), which was recently shown to be beneficial for in vivo optical imaging.

**Conclusions:**

A combination of PS with fluorescence in visible and NIR-I spectral ranges and, NIR-II fluorescent dye allowed us to obtain PS nanoformulation promising for see-and-treat PDT guided with visible-NIR-SWIR fluorescence imaging.

## Background

Photodynamic therapy (PDT) is a minimally invasive treatment method that selectively eliminates malignant tumor tissue through light-induced generation of the cytotoxic reactive oxygen species (ROS) by a tumor targeting PDT agent (photosensitizer, PS) [1–3]. The main advantages of PDT, as compared to more conventional methods of treatment (e.g., chemotherapy and radiation therapy), are high tumor specificity, low morbidity and traumaticity to healthy tissues, which result in a possibility of multiple repeats without a significant damage to the organism and an efficiency against tumors of various histogenesis [2]. Most of PS molecules fluoresce under excitation with light; fluorescence imaging can be employed in PDT to evaluate PS biodistribution and assess its intratumoral accumulation before the therapeutic light application [4–6]. However, excitation of the PS during assessment of its biodistribution through fluorescence imaging results in the premature photobleaching of PS and can cause toxicity to healthy tissues. An involvement of near infrared fluorescent dye (NIRFD) to tag PS moiety can allow for NIR fluorescence imaging guided delivery of PS to a tumor for phototherapy and/or fluorescence guided resection [7].

A combination of two separate moieties, NIRFD and PS, within the same molecular conjugate allowed Pandey’s group to introduce a “see-and-treat” concept in PDT [8, 9]. However, conjugation of PS and conventional NIR dyes has been shown to result in less efficient PDT, as it affects the PS pharmacokinetics and reduces PS efficacy through undesirable electronic excitation energy transfer (i.e., Förster Resonance Energy Transfer, FRET) between PS and NIRFD [9–11]. In other words, molecules with higher FRET show reduced singlet oxygen production and PDT efficacy. In particular, due to electronic excitation energy transfer between pi-electron systems of photosensitizer 2-(1-hexyloxyethyl)-2-devinyl pyropheophorbide-a (HPPH) and NIR fluorescent cyanine dyes, the amount of singlet oxygen produced by the HPPH-NIRFD conjugate upon excitation of the HPPH moiety is much lower than that produced upon excitation of unconjugated HPPH [11, 12]. As a result, the therapeutic application of HPPH-NIRFD conjugates requires a significantly higher dose than that of HPPH; this therapeutic dose is also much higher than the necessary imaging dose. The results obtained from a series of HPPH and cyanine dye conjugates suggest that the orientation of two chromophores and length of linker between them effect FRET efficiency and make significant difference for in vivo PDT efficacy [9, 10].

**Scheme 1:**
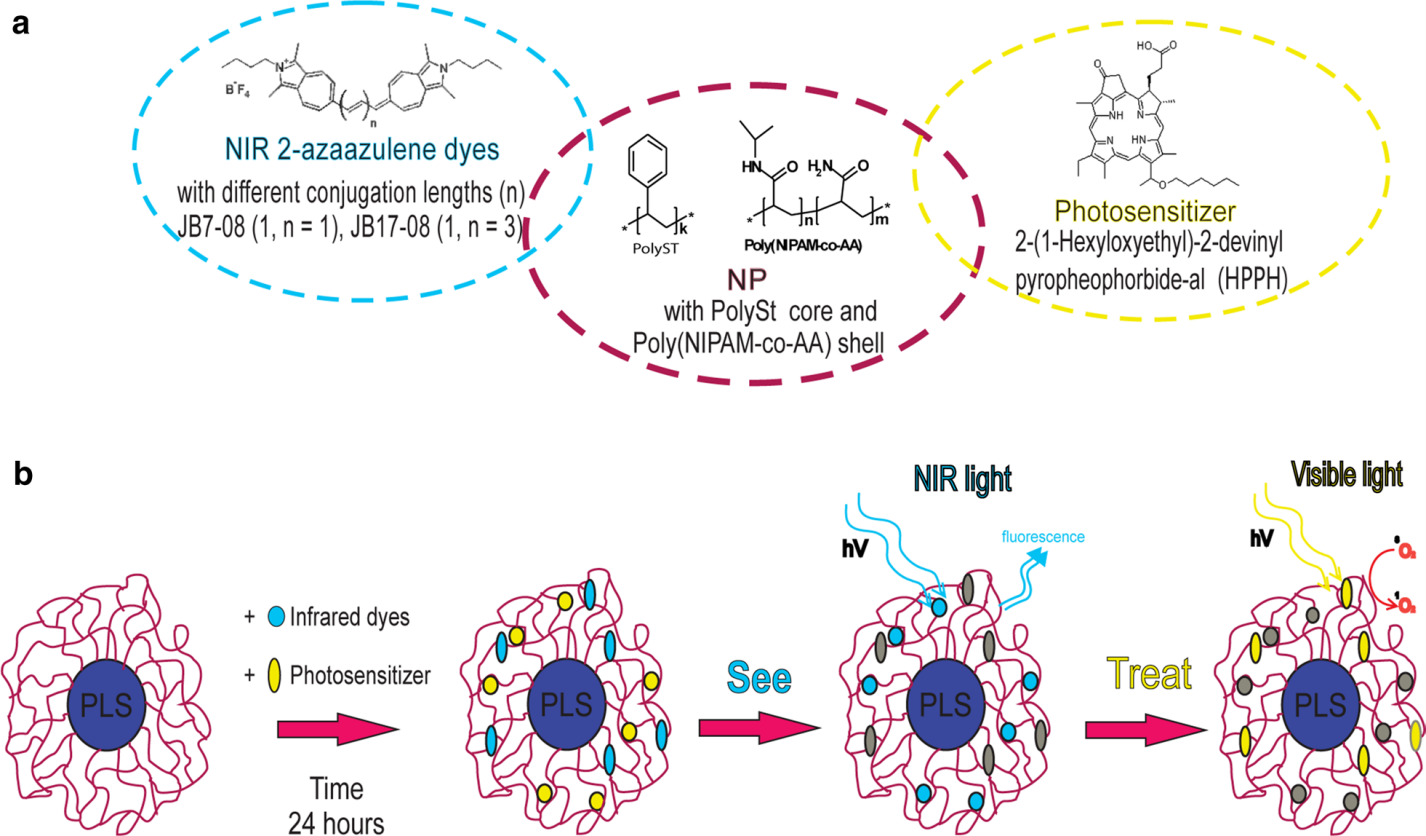
**a** chemical structures of NIRF dyes (left, circled by blue dashed line), polymers that form polySt-poly(NIPAM-*co*-AA) NPs (central, circled by purple dashed line) and photosensitizer (right, circled by yellow dashed line). **b** scheme illustrating preparation and application of nanoformulations for see-and-treat photodynamic therapy

Consecutively, Kopelman’s and Pandey’s groups introduced polyacrylamide (PAA) NPs as PDT drug delivery nanovehicle [13]. In this way, NPs were synthesized first and then the hydrophobic PS, dispersed in the water suspensions of NPs, diffused into the porous PAA NPs. This novel approach, in which the PS is adsorbed by the surface of the pre-formulated NPs (“post-loaded”), was identified as a promising method for loading PAA-based NPs with hydrophobic PS to achieve effective PDT in vitro and in vivo. It should be noted that an entrapment of hydrophobic PS by water dispersible NPs can enhance bioavailability and increase PS accumulation in the tumor due to the “Enhanced Permeability and Retention” (EPR) effect [14, 15]. The poor lymphatic drainage system in tumors allows for high fluid retention in the tumor interstitial space, which helps to retain nanoparticles and macromolecular objects within tumor, and limits an extravasation of these objects into normal tissue [16]. Kopelman’s and Pandey’s groups reported the use of a PAA NPs based nanoformulations (nanoconstructs combining both the PS (HPPH) and NIR fluorophore) [17]. After post-loading, both (NIRFD and PS) moieties were greatly retained in the NPs (as confirmed by the release kinetics study) that provided served as constructs for non-invasive detection of tumors and delineation of the tumor margins by NIR fluorescence imaging, in combination with PDT. However, the efficacy of PDT produced by PS/NIRFD post-loaded poly-AA NPs is decreased by the energy transfer between PS and NIRFD [13]. In addition to the conventional NIR-I window, other optical windows have recently been identified in NIR-II region (or SWIR, as defined by the manufacturers of imaging cameras for ~ 1000–1700 nm range) for in vivo optical imaging [18–20]*.* The reduced tissue scattering and autofluorescence in SWIR spectral region results in a possibility to achieve bioimaging of deeper tissues with better resolution [21–23]. Thus, use of PS-NIRFD combination, which includes NIRFD emitting in SWIR range, can be beneficial for fluorescence imaging guided PDT. Herein, we report polymeric nanoparticles (NPs) with polySt core and shell of co-polymer of *N*-isopropylacrylamide and acrylamide, [polySt-poly(NIPAM-*co*-AA)] as nanoplatform for NIRFD/PS combination (Scheme 1). A core–shell structure of these nanoparticles allows to entrap hydrophobic molecules added to the nanoparticle suspension [24]. Aiming to NIR/SWIR fluorescence imaging-guided PDT, core–shell NPs in this work were post-loaded with HPPH and NIRFD with fluorescence in NIR-SWIR region. Use of core–shell nanoparticles, where PS and dye molecules were separately post-loaded, along with application of SWIR fluorescent dyes, resulted in less efficient energy transfer between the two chromophores (PS and dye) and, correspondingly, lesser reduction in singlet oxygen generation. A shift of the NIRFD absorption to longer wavelengths was demonstrated to result in the reduced efficiency of undesirable electronic excitation energy transfer between PS and NIRFD, which diminishes the efficacy of PDT with NIRFD–PS combination. Thus, use of SWIR emitting dyes allows for SWIR fluorescence imaging with higher contrast, sensitivity and penetration depth in comparison with conventional NIR fluorescence imaging.

## Materials and methods

### Materials

Styrene (ST, Ukraine) of p.a. quality was purified via standard method directly before polymerization. *N*-isopropylacrylamide (NIPAM, Sigma-Aldrich Inc.), *N*,*N*′-methylenebisacrylamide (BIS, Sigma-Aldrich Inc.), potassium persulfate K_2_S_2_O_8_ (KPS, Ukraine), sodium phosphate monobasic dihydrate NaH_2_PO_4_ × 2H_2_O (KPS, Ukraine), were of reagent grade and used without further purification. HPPH (2-[1-hexyloxyethyl]-2-devinyl pyropheophorbide-alpha) was purchased from Raybiotech, China. Phosphate-buffered saline with pH 7.4 (PBS) was purchased from Gibco Life Technologies (AG, Switzerland). 9,10-anthracenediyl-bis(methylene)dimalonic acid (ABDA), anionic surfactant sodium dodecyl sulfate (SDS), chloroform, and dimethyl sulfoxide (DMSO) were purchased from Sigma Aldrich (Saint Louis, MO). Two 2-azaazulene polymethine dyes with different conjugation lengths (JB7-08 and JB17-08) were synthesized as described previously [25].

### Synthesis of NPs

Polymeric nanoparticles with polystyrene core and shell of co-polymer of *N*-isopropylacrylamide and acrylamide, polySt-poly(NIPAM-*co*-AA), were synthesized by microemulsion polymerization, with modification of the method described previously [26]. First, polySt core nanoparticles with 10% wt content of PNIPAM were prepared. Briefly, 0.1 g of NIPAM, 0.1 g of SDS and 0.005 g of NaH_2_PO_4_ × H_2_O were dissolved in 45 g of H_2_O. After the temperature increased to 60 °C, 1 g of styrene was added dropwise during 30 min at vigorous stirring. The mixture was stirred at 1200 rpm and Ar was bubbled into the mixture for 30 min. After the temperature increased to 70 °C, 0.08 g of K_2_S_2_O_8_ dissolved in 5 ml of H_2_O was injected to initiate the polymerization. Second, poly(NIPAM-*co*-AA) shell was layered onto the polySt core. For this purpose, aqueous solution of monomer NIPAM (1.63 g), AA (0.17 g) and cross-linker *N*,*N*′-methylene bisacrylamide (BIS) (0.18 g) was added to the 4 ml of H_2_O using a syringe. The reaction was allowed to continue for 4 h at 70 °C. The mixture was cooled to room temperature and dialyzed during 74 h using cellulose membrane with MWCO 3500 Da. As a result, suspension of the polySt-poly(NIPAM-*co*-AA) nanoparticles were fabricated with core–shell structure that is clearly revealed in the Transmission Electron Microscopy (TEM) images (Additional file 1: Figure S1). For NPs to be imaged with TEM, phosphotungstic acid was first added (2%) to the NPs suspension as a contrast agent. Second, the TEM samples were prepared: 10 µL of NPs suspension was dropped onto carbon support film stabilized with formvar. The TEM imaging of the polystyrene-NIPAM-*co*-AA nanoparticles were performed using HT 7700 (Hitachi Ltd., Tokyo, Japan) transmission electron microscope. The size and polydispersity index (PDI) of NPs were determined by the dynamic light scattering technique (DLS) using a particle size analyzer (Zetasizer Nano ZS, Malvern).

### Preparation and characterization of NFs

To prepare the nanoformulations, NPs_D2 were post-loaded with HPPH and NIRFDs. Briefly, 10 µl of the HPPH stock solution (1 mM in DMF) and 7 µl of the NIRFDs (JB7-08 or JB17-08) stock solution (1 mM in DMF) were consecutively added to 90 µl of NPs_D2 suspension (2% w/v). The resulted suspension was carefully mixed by micropipette and kept overnight in dark at room temperature. Next day, the sample volume was increased to 2 ml by adding distilled water and, after careful mixing, the NF were yielded, containing 5 µM of HPPH, 3.5 µM of NIRFDs (JB7-08 or JB17-08) and 0.1% w/v of NPs_D2. For evaluation of singlet oxygen generation and in vivo experiments, the NF with higher concentration of dyes and PS (7 µM and 10 µM, respectively) and NPs_D2 (0.3% w/v) were prepared.

The absorption spectra of the solutions and NFs were acquired using a spectrophotometer LAMBDA 750 UV/VIS/NIR (PerkinElmer). Fluorescence spectra in visible and NIR ranges were obtained using HORIBA Fluorolog-3 spectrofluorometer coupled for NIR-SWIR range with iHR320 spectrometer equipped by HORIBA DSS-IGA010L point detector. To excite NIRFD fluorescence, a collimated beam from the fiber-coupled laser diode emitting at 808 nm (QSP-808-4, QPhotonics) was introduced inside the sample chamber of the Fluorolog-3 spectrofluorometer and aligned to the sample cuvette to excite fluorescence from sample solutions and NFs.

The dialysis of nanoformulations was performed using Float-A-Lyzer G2 (300 kDa) dialysis device (Spectrum Lab, Inc., USA). The NF samples were dialyzed against distilled water for 24 h at room temperature.

### Singlet oxygen measurements

Chemical oxidation of ABDA was utilized to characterize the singlet oxygen generation efficiency of free and nanoformulated PS. ABDA is known to form the corresponding endoperoxide by singlet oxygen bleaching [27]. In this study, samples of NF with the same HPPH concentration (10 μM) and with or without NIRF dyes (7 μM) were mixed with 0.06 mM ABDA in PBS. An absorbance decrease of ABDA at 380 nm was measured as a function of time on the LAMBDA 750 UV/Vis/NIR Spectrophotometer, excited by the nanosecond Nd:YAG pulsed laser at 532 nm (LS-2137, Lotis TII) and 10 Hz repetition rate.

Singlet oxygen generation was also evaluated directly via its luminescence (phosphorescence) emission peaked at 1270 nm [28, 29]. A Fluorolog-3 spectrofluorometer equipped with an infrared spectrometer (iHR320, Horiba) was employed to detect singlet oxygen emission. Singlet oxygen phosphorescence was detected by the thermoelectrically cooled NIR-PMT detector (H10330B-75, Hamamatsu) of the iHR320 spectrometer set to 1270 nm. Decays of singlet oxygen phosphorescence under pulsed laser excitation were recorded by the Digital Phosphor Oscilloscope (TDS 3034C, Tektronix) coupled to the output of the NIR PMT. The sample suspensions were placed in quartz cuvettes and excited by the nanosecond Nd:YAG pulsed laser at 532 nm (LS-2137, Lotis TII) and 10 Hz repetition rate.

### Cell culture, cellular imaging and PDT in vitro

HeLa cells were grown in Advanced DMEM (Life Technologies) cell medium, supplemented with 2.5% fetal calf serum (FBS) (Sigma, St. Louis, MO), 1% glutamax (Life Technologies), 1% Antibiotic Antimycotic Solution (Sigma) at 37 °C in a humidified atmosphere containing 5% CO_2_. Prior to the treatment with NF containing HPPH and JB7-08 orJB17-08, cells were seeded into glass-bottom dishes (MatTek, Ashland, MA). A stock solution of HPPH and NIRFD were prepared in DMF. The final concentration of NIRFD and HPPH in NF were 14 µM and 20 µM respectively in 150 µl final solution of PBS. PDT of the cultured cells was initiated by optical excitation of the HPPH within NF. Cells were incubated in the medium with the NF overnight, then thoroughly washed and subsequently irradiated for PDT treatment by 405 or 640 nm lasers while scanned in zoomed imaging mode of the laser scanning confocal microscope (Nikon A1, Japan). Following the laser irradiation (at 405 or 640 nm), medium was replaced with the fresh one and cells were incubated for 6 and 24 h at 37 °C. At the next step, for viability assessment, the cells were incubated for 1 h in a serum-free MEM medium containing 500 nM of propidium iodide (PI). After this, cells were washed and then imaged in the confocal microscope. A 561 nm laser was used for the detection of PI fluorescence signal.

### Animal studies

The BALB/c nude mice were obtained from the Guangdong Medical Laboratory Animal Center (Guangdong, China). Animals were kept at aseptic conditions in a small animal facility. Tumors were generated by subcutaneously injection of 10^7^ HeLa cells in 50 µl of PBS onto the back of mice. The imaging studies were performed when the tumor volumes reached about 100 mm^3^. Prior to imaging, male mice (6 weeks old, 20 ± 2 g) were anesthetized with 5% chloral hydrate (0.06 ml per gram of mouse weight) by intraperitoneal injection. After that, 0.1 ml of NF (contained 0.4 mg kg^−1^ HPPH and 0.2 mg kg^−1^ NIRFD) were injected intratumorally. The biodistribution of HPPH and NIRFD was assessed by fluorescence imaging at different time points post injection (1; 3; 6; 24; 48; 60 h). The mice were sacrificed after 60 h imaging point and major organs (spleen, liver, heart, lungs, skin, kidneys) and tumor were immediately extracted and imaged to assess the distribution of PS and NIRFD*.*

Fluorescence imaging of HPPH was performed with the camera Nikon DS-FI2. NIR camera (Xeva-1.7-320, Xenics, Belgium) equipped with focusing optics (TEC-M55MPW, Computar, USA) was used to image the NIR-SWIR fluorescence signal from NIRFDs. In HPPH imaging, the lamp Nikon Intensilight C-HGFI with bandpass optical filter (620 ± 10 nm) was used as an excitation source and fluorescence images of PS were acquired using another band-pass filter (700 ± 17.5 nm). Both filters were purchased from Edmund Optics (USA). For excitation of NIRFD emission, fiber-coupled laser diode at 808 nm (QSP-808-4, QPhotonics, USA) powered with the laser power source (Laser Source 4308, Arroyo Instruments, USA) was employed. For acquisition of in vivo and ex vivo images in NIR-SWIR range, an 850 nm longpass filter (Edmund Optics, USA) was used.

## Results and discussion

In this study, polySt-poly(NIPAM-*co*-AA) core–shell nanoparticles of two different sizes (NPs_D1 and NPs_D2) were utilized to combine NIRFD and PS into one NF for FI guided PDT. Two 2-azaazulene dyes with different length of polymethine linkers(JB7-08 and JB17-08 [25]), were chosen as NIRFD and 2-[1-hexyloxyethyl]-2-devinyl pyropheophorbide-a (HPPH) was employed as PS. The core–shell structure of the synthesized NPs, which can be visualized by loading with TEM contrast agent (Additional file 1: Figure S1a), allows for loading with NIRFD [13] and other small molecules (e.g., HPPH). According to results of DLS measurements, hydrodynamic diameters of two batches of polySt-poly(NIPAM-*co*-AA) nanoparticles (NPs_D1 and NPs_D2) are ~ 170 and 220 nm at room temperature (~ 20 °C) respectively. It should be noted that the difference between DLS and TEM results can be explained by that the NPs during TEM imaging are on the surface but not in suspension. Drying of the NPs suspension on the support causes their poly(NIPAM-*co*-AA) shell to shrink.

**Fig. 1:**
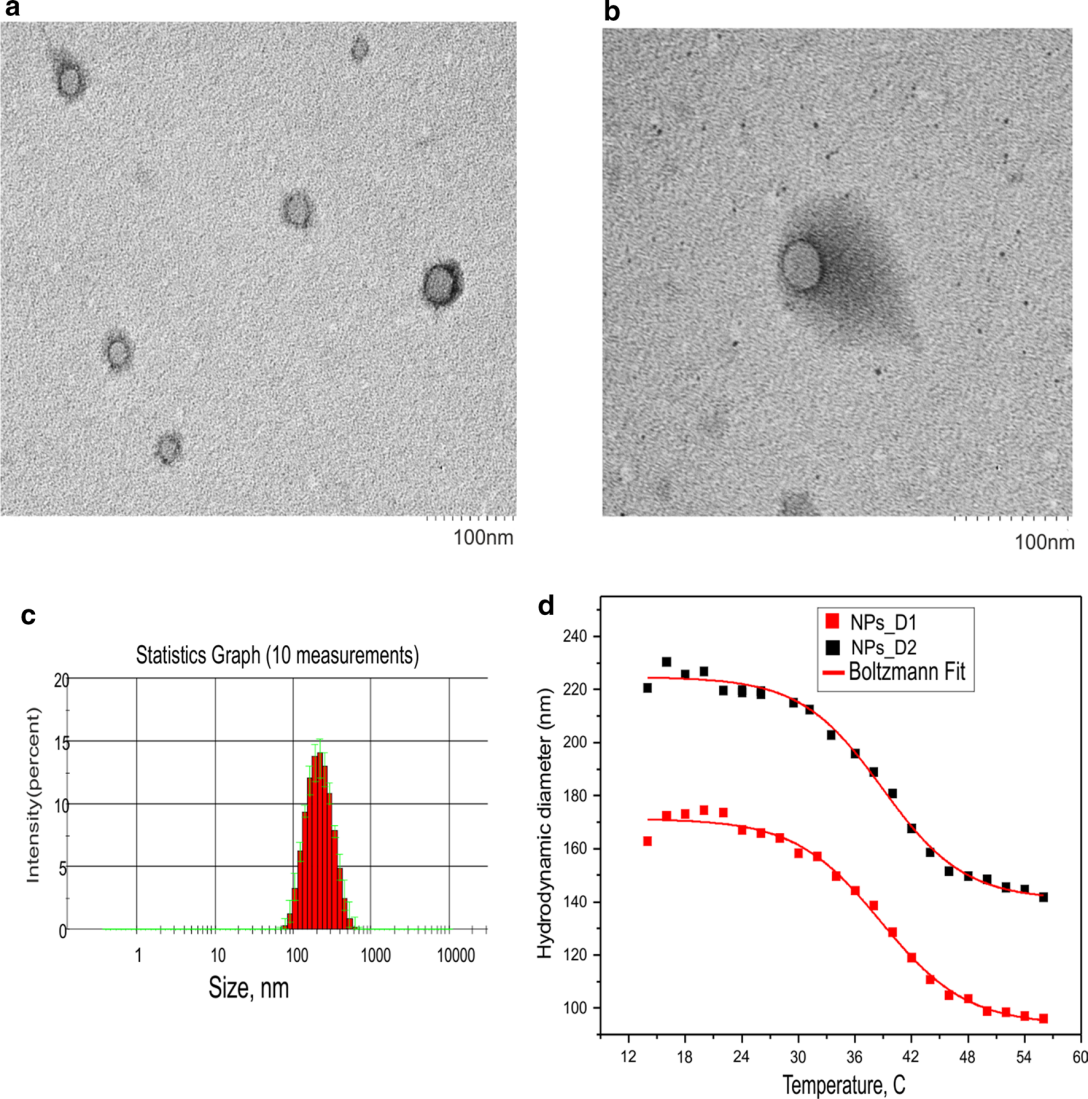
Size distributions on NF **a**,** b** transmission electron microscopy (TEM) images of polySt-poly(NIPAM-*co*-AA) core–shell NPs contained PS (10 µM HPPH) and NIRFD (7 µM JB17-08); **c** NPs size distribution determined by DLS. **d** dependence of NPs size on temperature, determined by DLS

PNIPAM is known to experience a conformational transition at lower critical solution temperature (LCST) T^0^ = 32 °C. Value of T^0^ can be shifted to 34–42 °C by copolymerization of NIPAM with more hydrophilic monomers, e.g. acrylamide (AA) [30]. In the developed NPs, shell is of poly(NIPAM-*co*-AA) copolymer, which makes NPs thermoresponsive. Changes in the NPs size resulting from the conformational changes of the copolymer shell induced by temperature rise were measured by DLS (Fig. 1c). The major size change was determined to occur at ~ 39 °C both for NPs _D1 and NPs_D2, suggesting that LCST of poly(NIPAM-*co*-AA) in the shell of NPs is close to this value.

Transition electron microscopy (TEM) images of the obtained NF (NPs post-loaded with PS and NIRFD) are shown in Fig. 1, clearly revealing the same core–shell morphology as blank NPs, with core size of ~ 30 nm. It should be noted that no notable difference can be found when comparing TEM images of blank NPs_D2 and that of HPPH/NIRFD loaded NF, both with contrast agent (Additional file 1: Figure S1a and Fig. 1a) and without it (Additional file 1: Figure S1b, c). Moreover, no noticeable difference between NPs_D2 and NF was also observed in DLS measurements (data not shown).

**Fig. 2:**
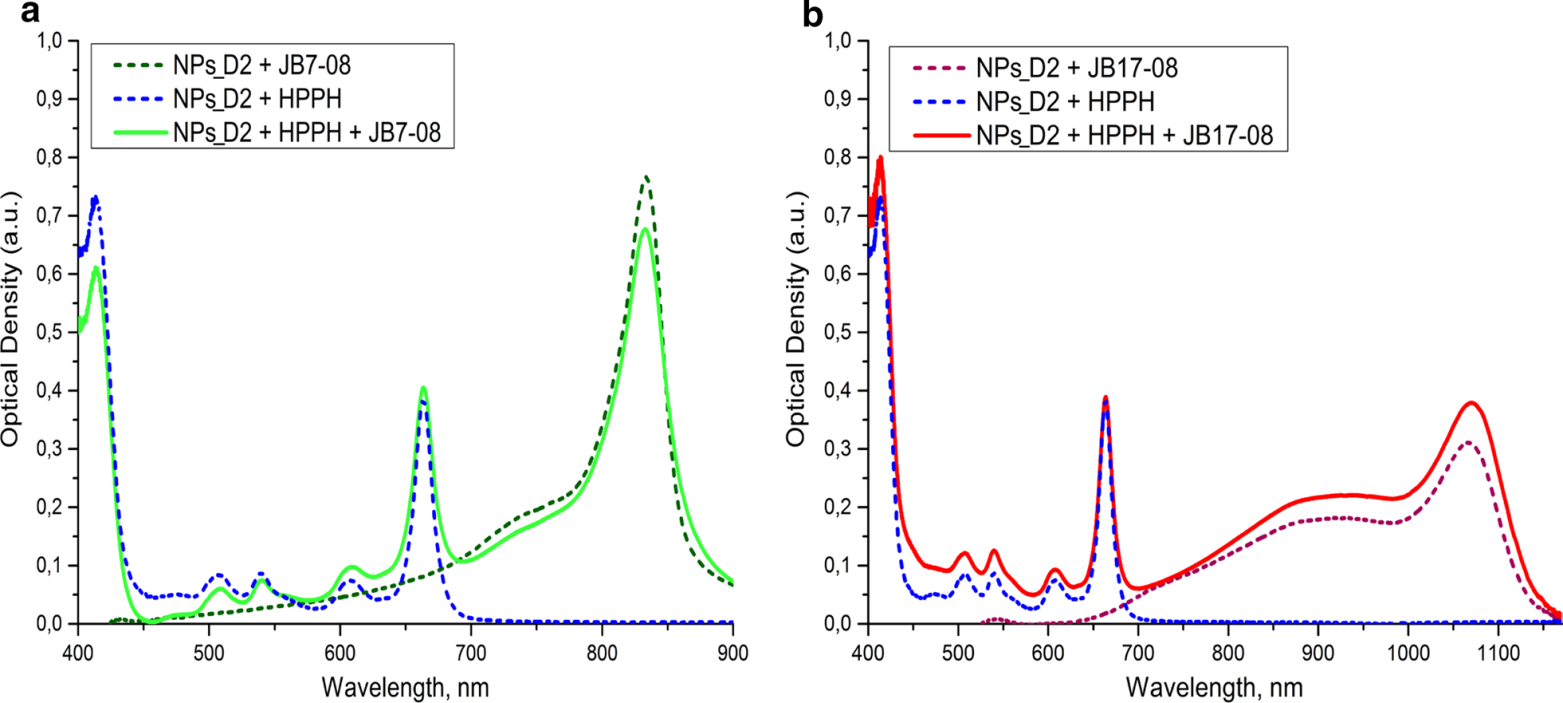
Absorption spectra of NF: **a** NPs_D2 post-loaded with HPPH alone or with JB7-08. **b** NPs_D2 post-loaded with HPPH alone or with JB17-08. Spectra were acquired 24 h after addition of HPPH and NIRFDs to water suspension of polySt-poly(NIPAM-*co*-AA) NPs

The absorption and fluorescence spectra of NF [NPs_D2 post-loaded with HPPH and/or NIRFD (JB7-08 and JB17-08)] are shown in Fig. 2. Absorption spectra reveal characteristic Soret and Q bands, which are associated with the presence of the HPPH moiety (corresponding peaks at ~ 408 and ~ 660 nm), as well as long-wave absorption by the NIRFD moiety. The fluorescence quantum yield for JB17-08 in organic solution is known to be around 0.05, which is considered as extraordinarily high for organic dyes emitting beyond 1000 nm [25]. Though JB7-08 and JB17-08 are virtually non-fluorescent in aqueous solutions, their fluorescence is restored through post-loading to the polymeric matrix of PS-PNIPAM nanoparticles [24]. As a result, the dye-loaded nanoparticles exhibit fluorescence in ~ 900–1100 nm spectral range under excitation at ~ 800 nm [31]. These spectral characteristics suggest that JB 7-08 and JB17-08 dyes are promising candidates as imaging agents co-loaded with HPPH in PAA-based NPs.

**Fig. 3:**
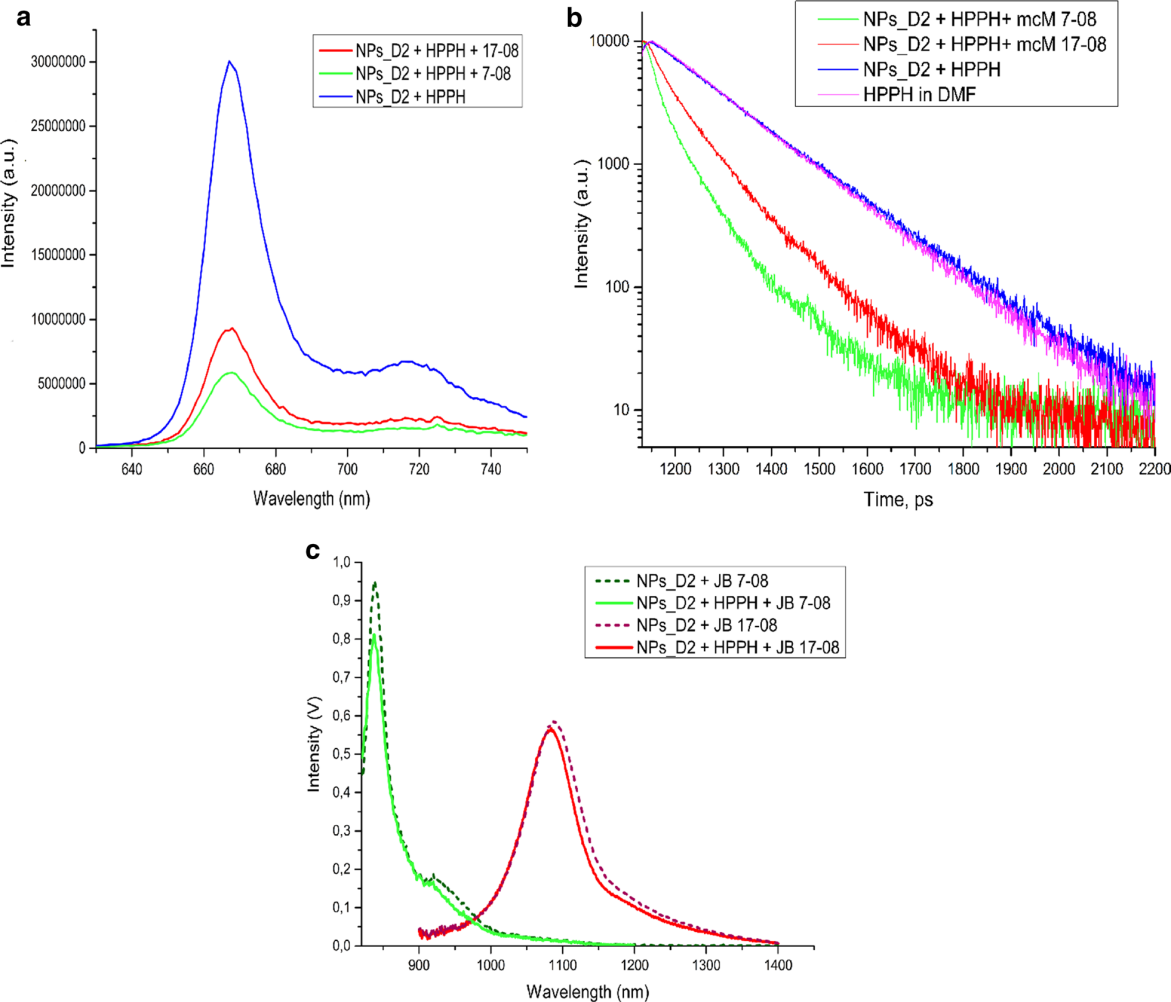
Fluorescence spectra of polySt-poly(NIPAM-*co*-AA) NPs post-loaded with HPPH and dyes. **a** HPPH fluorescence emission (excitation at 400 nm). **b** Fluorescence decays for HPPH in organic solvent (DMF) or in NF, post-loaded alone or with two different NIRFDs. **c** fluorescence of NIRFDs post-loaded to NF alone or in combination with HPPH (excitation at 808 nm)

Figure 2 shows that co-loading of NIRFD with HPPH does not noticeably affect the absorption of HPPH in nanoparticles, whilst spectra of NIRFD are slightly changed when dyes are co-loaded with HPPH. This change is apparently associated with the reduced interaction between NIRFD moieties, when they are co-loaded with HPPH, and corresponding increase in the monomeric dye absorption along with a decrease in the aggregation-related short-wavelength shoulder [18, 32]. It should be noted that since the absorption of JB7-08 is spectrally closer to the HPPH absorption than the absorption of JB17-08 (Fig. 2b), it apparently overlaps more with HPPH fluorescence emission. This can lead to more efficient transfer of electronic excitation energy between HPPH and JB7-08 and, as a result, lower intensity of HPPH fluorescence in NF with JB7-08 than in NF with JB17-08. We have acquired the fluorescence spectra of HPPH and NIRFDs in free state and after loading to polySt-poly(NIPAM-*co*-AA) NPs and found that the HPPH fluorescence intensity is noticeably higher for HPPH/JB17-08 NF than for HPPH/JB7-08 NF, while it is the highest for HPPH loaded alone (Fig. 3a). This observation suggests that NIRFDs, co-loaded together with HPPH to NPs, work as acceptors of the HPPH electronic excitation energy. Measurements of the fluorescence decays revealed that while fluorescence of HPPH loaded to NPs alone has lifetime of ~ 7.6 ns, which is close to that from a free HPPH in organic solvent (~ 7.3 ns), it shortens when HPPH is co-loaded to NPs together with NIRFDs (Fig. 3b). Moreover, as can be seen in Fig. 3b, HPPH fluorescence lifetime is shorter in NF with JB7-08 (~ 2.5 ns) than in NF with JB17-08 (~ 4 ns), confirming that a higher decrease in HPPH fluorescence intensity for HPPH/JB7-08 NF is associated with more efficient energy transfer from HPPH to NIRFD.

It is important to emphasize that the HPPH fluorescence intensity in NF depends only on the type of the added NIRFD. We have studied the dependence of HPPH fluorescence intensity on different sizes of nanoparticles and verified (through the use of NPs_D1 or NPs_D2) that the size of the nanoparticles does not noticeably affect the fluorescence intensity of HPPH: HPPH fluorescence intensity for NPs_D1 were the same as that for NPs_D2, but fluorescence of HPPH is higher—with the NIRD JB-17-08. We have also compared the fluorescence from HPPH in nanoparticles co-loaded with different concentrations of the JB-17-08 dye and found that intensity of HPPH fluorescence was higher at lower concentration of JB17-08 post-loaded to NF along with HPPH (see Additional file 1: Figure S2a, b).

On the other hand, it was also observed that loading of HPPH to NF does not significantly affect the fluorescence of the co-loaded NIRFD (when exciting at the wavelengths of NIRFD absorption), suggesting that HPPH in NF does not block binding of the dye molecules with NPs (Fig. 3c). Furthermore, in contrast to fluorescence of HPPH, fluorescence intensity of NIRFDs was found to be dependent on the size of nanoparticles they were loaded to. As one can see in Additional file 1: Figure S2c, the fluorescence intensity of JB17-08 is higher when it is post-loaded to the larger NPs (NPs_D2). This is apparently associated with the thicker shell of NPs_D2 nanoparticles that serves as a matrix for post-loaded NIRFD molecules and allows for more efficient post-loading than shell of NPs_D1. Due to more intense NIRFD emission from NPs_D2, these NPs were chosen for our further experiments on PDT and imaging in vitro and in vivo. It is also worth noting that the dependence of NIRFD fluorescence on the concentration of post-loaded dye shows deviation from linearity starting from 3.5–5 µM (for 0.1% w/v concentration of NPs, see Additional file 1: Figure S2c), at higher concentrations NIRFD fluorescence is quenched to some extent. In addition, we acquired the absorption and fluorescence spectra of NF before and after dialysis and did not find any significant changes in the spectral shape, while absorption and fluorescence intensities where somewhat decreased, pointing to a slight release of HPPH and NIRFD during dialysis (Additional file 1: Figure S3).

As a result, post-loading, when the PS is adsorbed by the surface of NPs, was identified as the promising method for loading poly-*N*-isopropylacrylamide-based NPs with hydrophobic PS to achieve effective PDT.

**Fig. 4:**
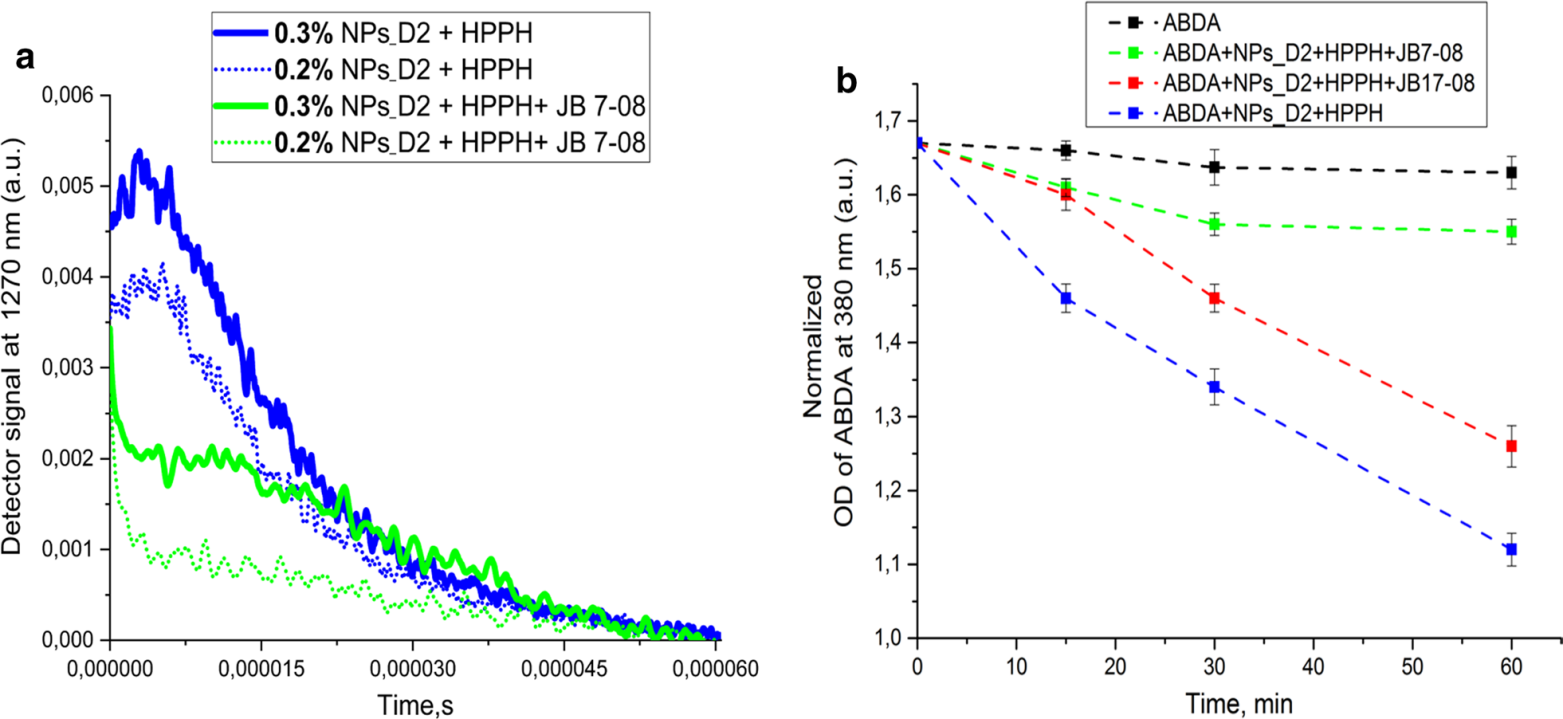
Evaluation of singlet oxygen production by the NF. **a** phosphorescence decays of singlet oxygen (at 1270 nm) sensitized by NF with HPPH only and HPPH/JB7-08 NF. Two concentration of NPs_D2 was used for preparation of each NF. **b** ABDA bleaching as a measure of the singlet oxygen generation by ABDA in water, and in suspensions of HPPH, HPPH/JB7-08 and HPPH/JB17-08 NFPPH loaded to NPs and NF

**Fig. 5:**
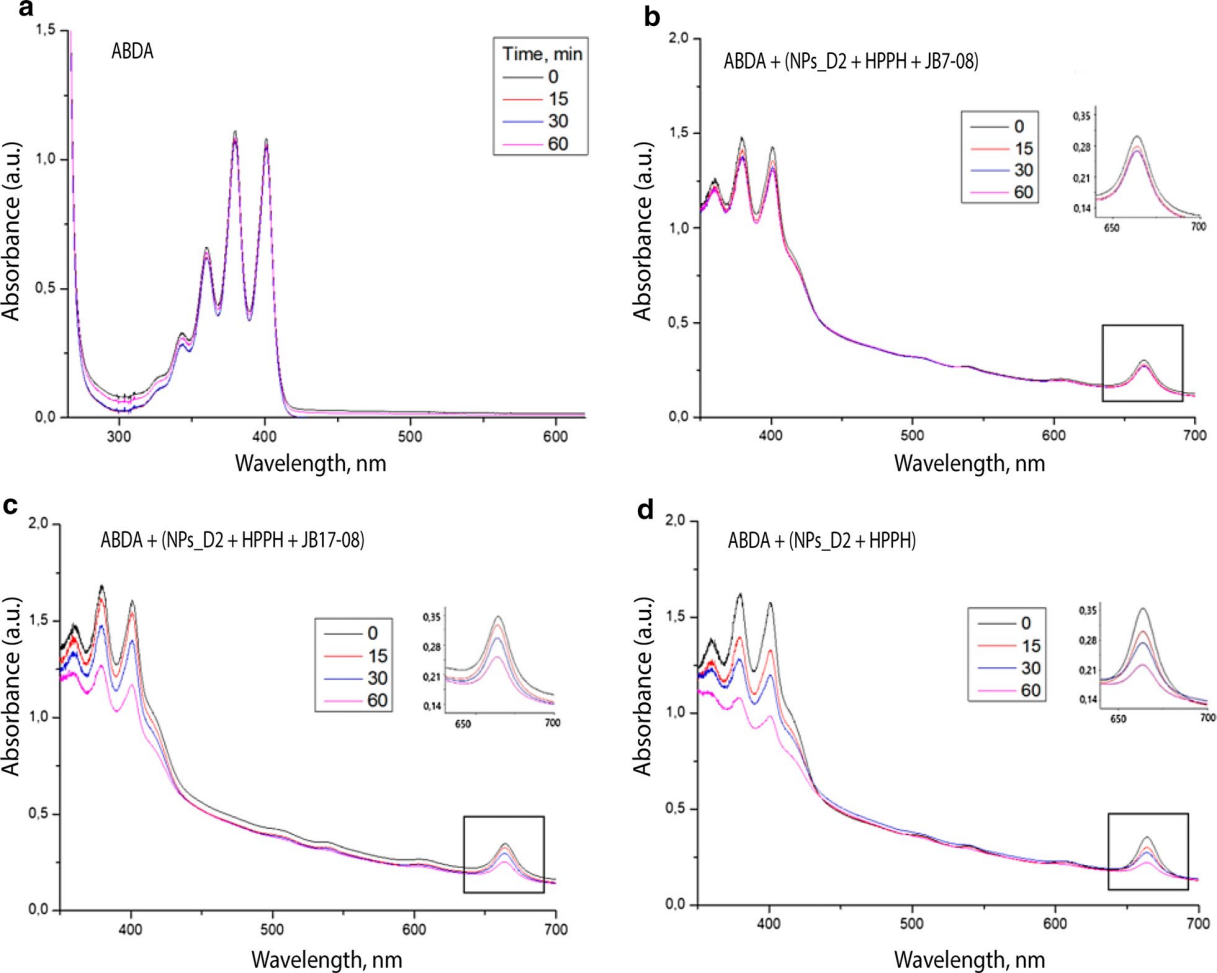
Changes in absorption of ABDA and HPPH under 532 nm laser irradiation. Samples were irradiated for 0, 15, 30 and 60 min. **a** free ABDA in PBS. **b** HPPH/JB7-08 NF suspension with added ABDA. **c** HPPH/JB17-08 NF suspension with added ABDA. **d** HPPH NF suspension with added ABDA

As HPPH fluorescence was less quenched in HPPH/JB17-08 NF than in HPPH/JB7-08 NF, which is apparently associated with less efficient drainage of HPPH electronic excitation in HPPH/JB17-08 NF, we further determined if this correlates with singlet oxygen production by HPPH in both NF. Singlet oxygen (^1^O_2_) is the major cytotoxic agent in PDT and the reaction between ^1^O_2_ and tumor tissues/cells determines the treatment efficacy [33, 34]. Among the approaches for singlet oxygen dosimetry, singlet oxygen luminescence dosimetry (SOLD) [34, 35] is considered to be a reliable direct ^1^O_2_ measurement method, utilizing the luminescence emission of ^1^O_2_ peaked at 1270 nm to evaluate the production of ^1^O_2_, which is relevant to PDT efficacy [15, 36]. Figure 5a shows decays of singlet oxygen phosphorescence produced by NPs_D2 post-loaded with HPPH or HPPH/JB7-08 nanoformulations under pulsed laser excitation at 532 nm. As one can see, NF containing HPPH alone does produce more singlet oxygen than HPPH/JB7-08 NF, which correlates with difference in HPPH fluorescence intensity from these two nanoformulations. It should be, furthermore, noted that, similarly to HPPH fluorescence intensity, ^1^O_2_ emission intensity is higher for higher concentration of nanoparticles (for the same amount of HPPH), which is apparently associated with lesser interaction (e.g., aggregation) between HPPH molecules, when more NPs are available for binding (post-loading) (Fig. 4a). Unfortunately, even if the ^1^O_2_ phosphorescence measurement is a reliable method that allows for direct evaluation of singlet oxygen production, it might have limited applicability in some cases. In particular, when trying to acquire ^1^O_2_ emission decay from HPPH/JB17-08 NF suspension, we have found that NIRFD fluorescence signal at 1270 nm is so high that it saturates the detector, making measurements of ^1^O_2_ emission impossible, even with time-resolved approach. In this regard, comparison between ^1^O_2_ production efficiency for HPPH/JB7-08 and HPPH/JB17-08 was performed using indirect measurements with singlet oxygen-sensitive analyze.

Specifically, production of singlet oxygen by NF was evaluated through monitoring the photo-oxidation of 9,10-anthracenediyl-bis (methylene) dimalonic acid (ABDA). When molecules of ABDA and singlet oxygen interact, the anthracene moiety undergoes a specific reaction to form a 9, 10-endoperoxide product. This singlet oxygen-specific reaction can be assessed spectrophotometrically to quantify singlet oxygen production by measuring the decrease in optical absorbance at 380 nm (the absorption peak of anthracene chromophore) [27, 36, 37].

Figure 4b shows ABDA bleaching (change of absorption at 380 nm with time under irradiation at 532 nm) for ABDA in water and when it is added to suspensions of HPPH, HPPH/JB7-08 and HPPH/JB17-08 nanoformulations (which contain the same amount of HPPH). As one can see, ABDA in HPPH/JB17-08 NF suspension bleaches faster than when added to HPPH/JB7-08 suspension, confirming that the HPPH/JB17-08 NF produces more singlet oxygen than the NF containing HPPH with JB7-08, though less than HPPH NF (HPPH alone post-loaded to NPs_D2). Again, it is worth noting that the singlet oxygen production efficiency in NF changes in the same way as intensity of HPPH fluorescence: it is the highest for HPPH NF, lower for HPPH/JB17-08 and the lowest for HPPH/JB7-08.

Figure 5 confirms this trend, showing changes in HPPH absorption in NF under irradiation. As the produced ^1^O_2_ also reacts with HPPH itself, rate of HPPH bleaching should correlate with the amount of the generated singlet oxygen. As can be seen in Fig. 5b–d, absorption peak of HPPH at ~ 665 nm decreases with time of irradiation and its rate is different: the highest for HPPH NF, lower for HPPH/JB17-08 NF and the lowest for HPPH/JB7-08 NF. It should be also noted that presence of ABDA in the suspension does not noticeably affect HPPH bleaching under 532 nm irradiation (Additional file 1 Figure S4). Figure 5a additionally illustrates stability of ABDA under 532 nm excitation in absence of HPPH, when ^1^O_2_ is not generated.

**Fig. 6:**
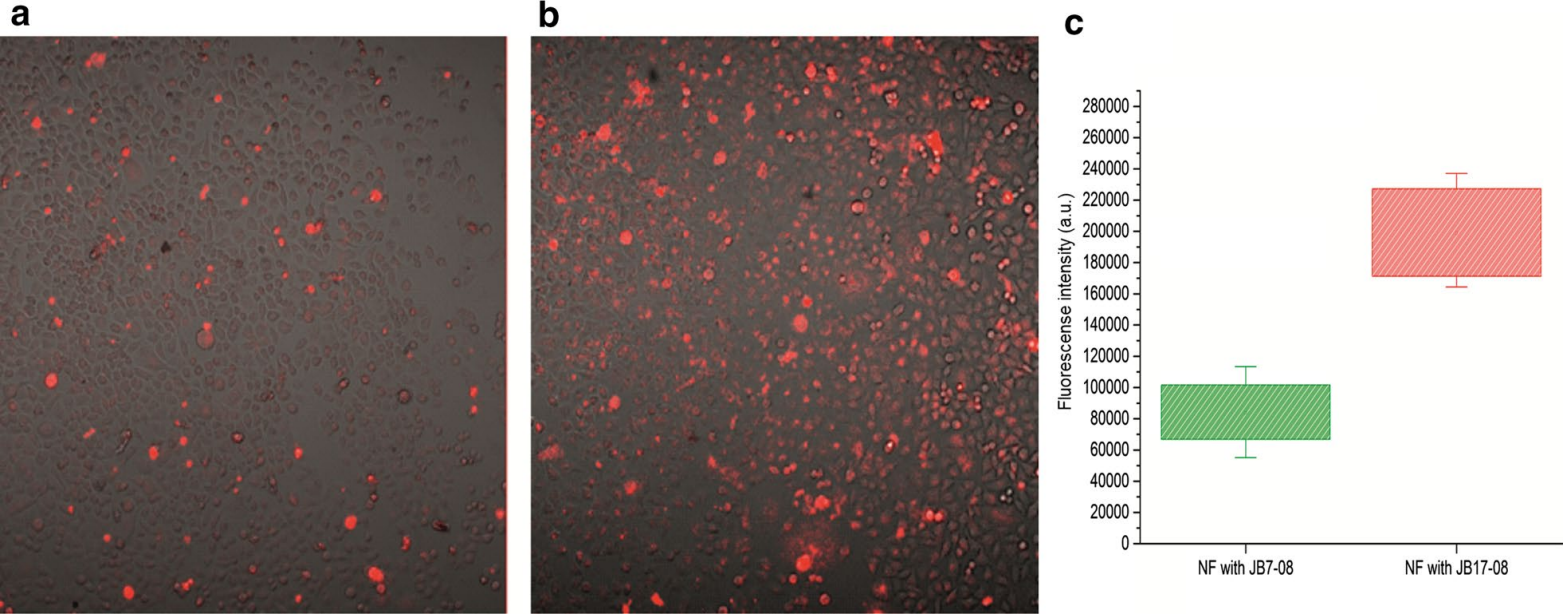
The confocal images of HeLa cells treated with HPPH/JB7-08 (**a**) and HPPH/JB17-08 (**b**) nanoformulations**.** Panel **c** shows histogram of HPPH fluorescence intensities in **a** and **b**

To assess cellular uptake and photodynamic effect for HPPH/NIRFD NF, HeLa cells were first incubated overnight with either HPPH/JB7-08 or HPPH/JB17-08 nanoformulations and imaged using laser scanning confocal microscope. Figure 6a, b shows merged transmission and HPPH fluorescence images of cells treated with NF. It is obvious that the level of HPPH fluorescence in Fig. 6a (cells incubated with HPPH/JB7-08 NF) is significantly lower than that in Fig. 6b (cells incubated with HPPH/JB17-08 NF); the difference is illustrated by histogram in Fig. 6c. This, again, suggests, that photodynamic action of HPPH/JB7-08 NF is lower than HPPH/JB17-08 NF.

To confirm that higher singlet oxygen production and HPPH fluorescence signal from cells treated with HPPH/JB17-08 NF (in comparison HPPH/JB7-08 NF) results in stronger photodynamic action, we have performed PDT in vitro followed by a live/dead cell imaging assay with propidium iodide (PI) imaging probe. PI is a fluorescent nucleic acid stain (excitation/emission peaks are at 536/617 nm) that can permeate only the damaged membranes and is used as a selective marker of necrotic or late apoptotic cells. Using this assay in fluorescence microscopy, one can distinguish dead or dying ones, exhibiting red fluorescence [38, 39].

**Fig. 7:**
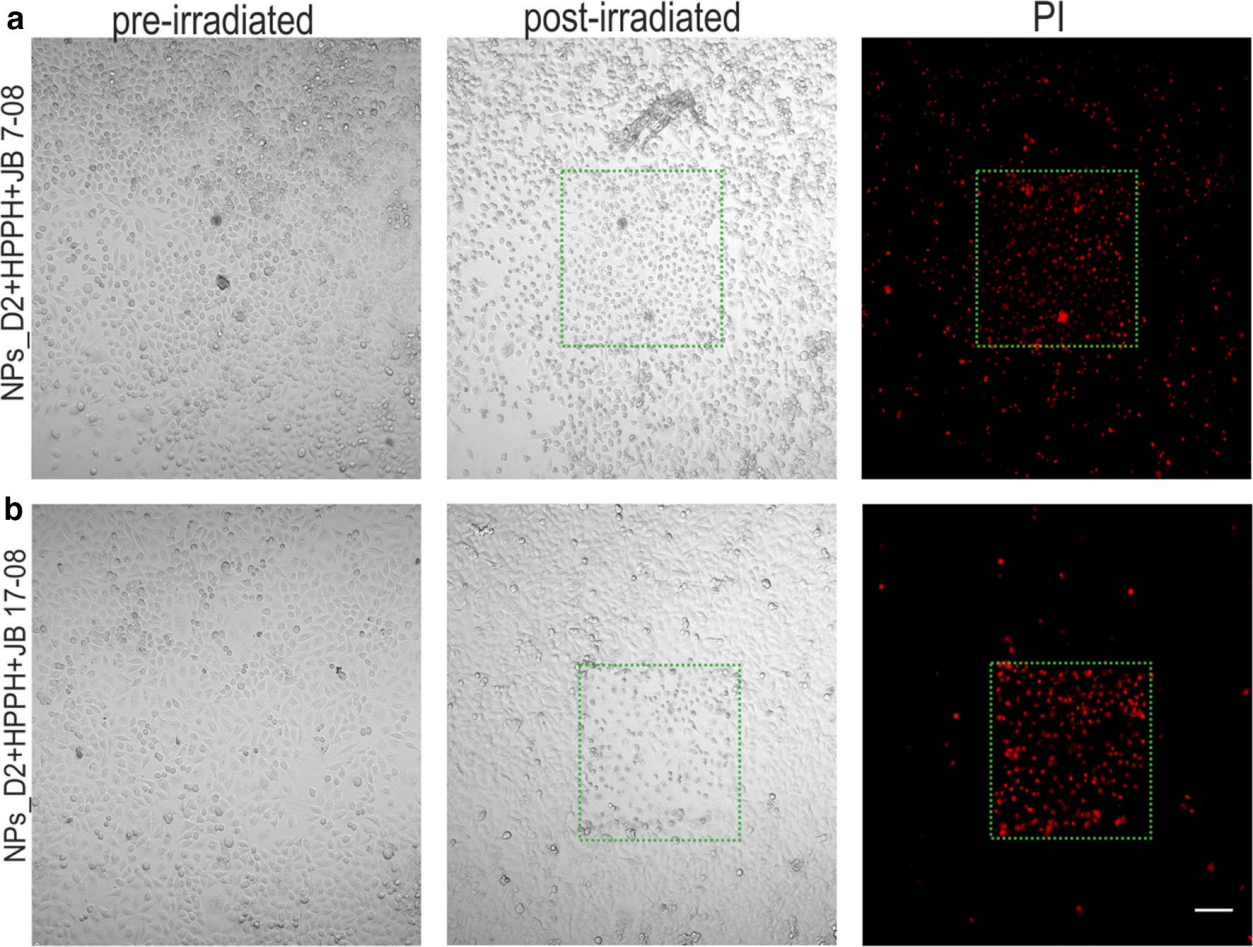
Visualization of the photodynamic effect produced in vitro by HPPH/JB7-08 (**a**) and HPPH/JB17-08 (**b**) nanoformulations. HeLa cells were treated overnight with NF, rinsed and irradiated in confocal microscope by scanning with 640 nm laser. 24 h post-irradiation, cells were stained with PI and imaged in confocal microscope to visualize irradiated area with necrotic cells displaying PI fluorescence. Transmission images of pre- and post-irradiated cells (left and central columns) and fluorescence images of post-irradiated cells (right column) are shown

**Fig. 8:**
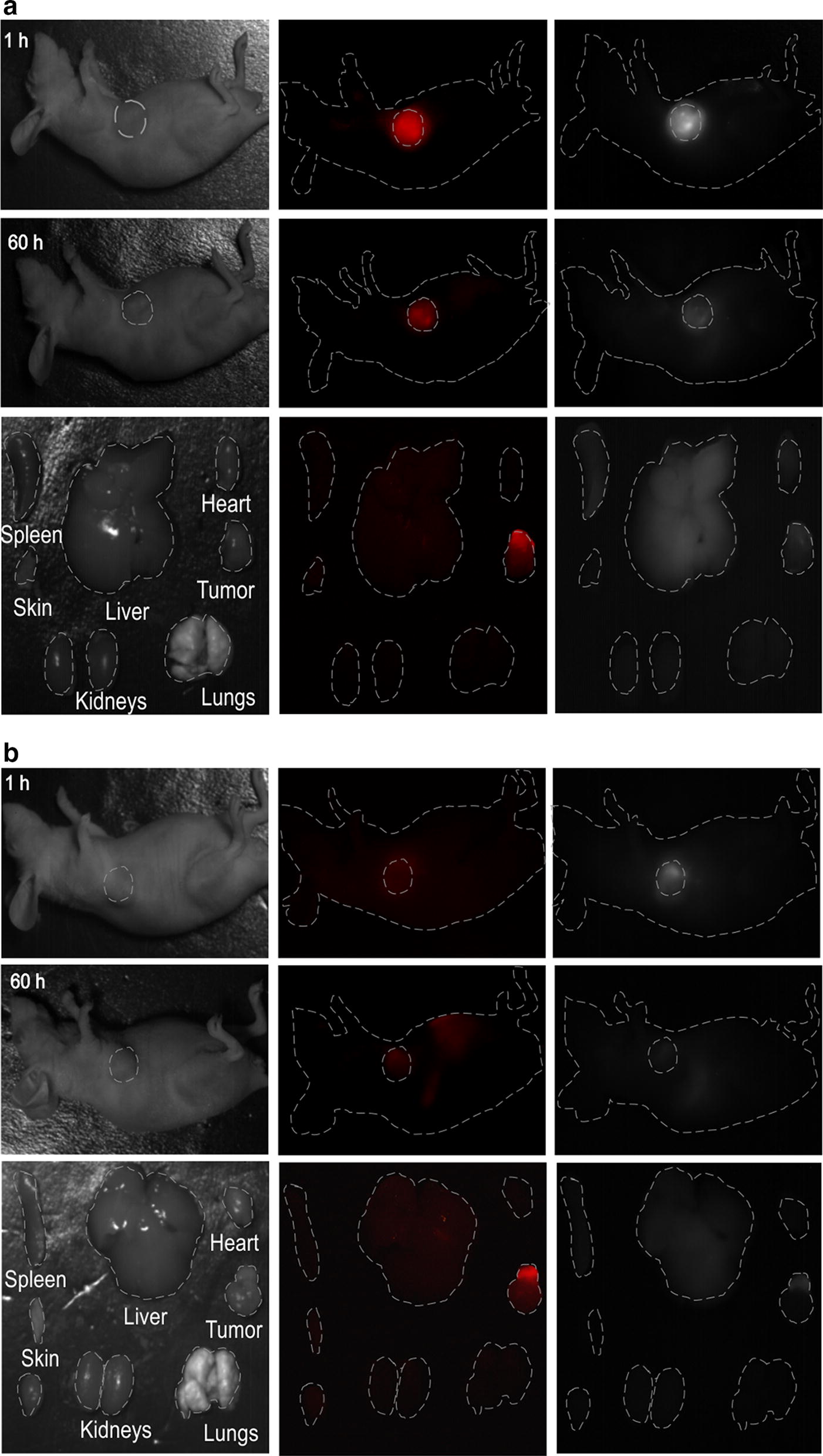
Bright field and fluorescence images of a nude mouse with a tumor at various time points (1 and 60 h) and major organs resected from mouse at 60 h post-injection with: **a** 200 µL of PBS with NF (0.3% NPs_D2 + 10 µM HPPH + 7 µM JB17-08). **b** 200 µL of PBS with HPPH and NIRFD (10 µM HPPH + 7 µM JB17-08)

Figure 7 clearly shows a dramatic difference in viability between cells, which were treated with HPPH/JB7-08 NF or with HPPH/ JB17-08 NF, and irradiated by scanning 640 nm laser. The irradiated (laser scanned) areas are of square shape and necrotic cells are much more evident in the area for cell dish treated with HPPH/JB17-08 NF than for dish treated HPPH/JB17-08 NF, as can be seen both in transmission and fluorescence channels. The 405 nm laser irradiation of cells treated with HPPH/JB7-08 NF or HPPH/JB17-08 NF also resulted in more efficient PDT effect for nanoformulation containing HPPH and JB17-08 (Additional file 1: Figure S5), confirming that use of NIRFD with absorption shifted towards longer-wavelengths relatively to PS absorption is beneficial for the PDT efficiency of PS/NIRFD formulation. At the same time, long-wavelength absorbing NIRFD can manifest NIR-II fluorescence, as JB17-08 does. To test the possibility of using HPPH/JB17-08 NF for NIR-II imaging guided PDT, we conducted experiments in vivo. HPPH/JB17-08 nanoformulation and HPPH/JB/17–08 mixture as a control were intratumorally injected into mice and fluorescence imaging (FI) was performed at certain intervals after injection (1, 6, 24, 48, 60 h). The results of the in vivo FI for 1 and 60 h are shown in Fig. 8. They clearly demonstrate the feasibility of simultaneous imaging of the spectrally distinguished fluorescence of HPPH and NIR-II fluorescent NIRFD from the same NF. Bright co-localized signals of red (HPPH) and NIR-II (JB17-08) fluorescence from the NF are striking in the tumor site at 1 h post-injection of HPPH/JB17-08 NF in PBS, and become less bright but still clearly visible at 60 h post-injection (Fig. 8a), in contrast to the control mice (injected with mixture of HPPH and NIRFD PBS suspension), where weaker signal of dye can be identified at 1 h post-injection but at 60 h post-injection only HPPH signal is seen. It is interesting to note the difference in biodistribution of HPPH and JB17-08 when they were injected in NF or in mixture.

Sixty hours after injection of HPPH/JB7-08 NF or HPPH/JB17-08 mixture and acquisition of the in vivo fluorescence imaging, the mice were sacrificed and their organs were resected and imaged immediately (representative images shown in Fig. 8). The ex vivo FI of the resected organs of mouse injected with NF revealed that HPPH was located mainly in tumor and could not be seen in kidney, liver, spleen, lungs, heart and skin (Fig. 8a). In contrast, when HPPH was injected together with NIRFD in a PBS suspension, a significant amount of its fluorescence could be found in liver, though signal from tumor was as bright as that from HPPH in NF (Fig. 8b). It should be noted that as HPPH is hydrophobic, it can form non-fluorescent nanocrystals in aqueous suspension, which can be converted to fluorescent molecular form through slow interaction with serum lipoproteins [40]. This could be a reason that HPPH fluorescence was not seen in tumor 1 h post-injection of HPPH/NIRFD mixture (unlike NIRFD fluorescence) but became visible 60 h post-injection (Fig. 8b). On the other hand, 60 h post-injection of JB17-08 in a PBS-based mixture with HPPH, SWIR fluorescence signal from NIRFD could not be found in any animal organs, suggesting that the dye was either degraded or excreted from the body. To the contrary, when JB17-08 was delivered to tumor in NF, SWIR fluorescence signal was still seen in the tumor, though a significant signal was also located in liver (Fig. 8a). The latter suggests that some amount of NIRFD can be separated with HPPH after intratumoral injection, excreted from tumor to the circulation (probably, as a part of degraded NF) and be consecutively filtered out of the circulation by liver. It is worth noting that though a full pharmacokinetics of HPPH/JB17-08 NF remains unclear, a possibility to track NF through fluorescence imaging both in visible and SWIR regions has been demonstrated in our work. This observation, along with the spectroscopy and in vitro PDT data, allows us to suggest that the developed nanoformulation is a promising step toward the SWIR imaging guided PDT.

## Conclusions

We report a multifunctional nanoplatform that provides optical (fluorescence) imaging and PDT. The synthesized polymeric nanoparticles with polystyrene core and poly(NIPAM-*co*-AA) shell can be post-loaded with PS (HPPH) and NIRFD with fluorescence in NIR-SWIR region to create a nanoformulation that possesses both PDT and NIR-II fluorescence imaging modalities. A shift of the NIRFD absorption to longer wavelengths was demonstrated to result in a reduction of the undesirable electronic excitation energy transfer between PS and NIRFD, enhancing the phototoxicity of NIR-II NIRFD–PS formulation in comparison with more conventional one that combines PS and NIR-I fluorescent dye. Furthermore, the in vivo imaging of tumored mice injected with NF carrying both HPPH and NIR-II (SWIR) fluorescent dye (JB17-08) was performed, illustrating a possibility to track this PDT active NF in vivo by SWIR fluorescence imaging, which provides higher contrast, sensitivity and penetration depth in comparison with conventional NIR fluorescence imaging. Studies are on the way to advance the proposed NF and achieve an enhanced PDT in vivo guided by SWIR fluorescence imaging.

## Supplementary information

**Additional file 1: Figure S1.** Transmission electron microscopy (TEM) images of NPs_D2 with (a) and without (b) TEM contrast agent (phosphotungstic acid); NPs loaded PS and NIRFD without contrast agent (c). **Figure S2.** Fluorescence spectra of NF: polySt-poly(NIPAM-co-AA) NPs loaded with HPPH and dyes. **a** Fluorescence of HPPH emission loaded to NPs_D1 and NPs_D2 (excitation at 400 nm). **b** Fluorescence of NPs_D2 loaded with HPPH only and with HPPH and different concentration of JB 17-08; **c** Dependence of fluorescence intensity of JB 17-08 loaded NPs_D1 and NPs_D2 on dye concentration. Fluorescence was excited at 808 nm. **Figure S3.** Absorption (a) and fluorescence spectra (b, c) of HPPH and JB17-07 loaded to NPs_D2 before (black) and after (red) dialysis. **Figure S4.** Changes in absorption spectra of HPPH loaded to NPs_D2 under 532 nm laser irradiation. Samples were irradiated for 0,15,30 and 60 min. **Figure S5.** Confocal microscopy images of HeLa cells pre-incubated with HPPH/NIRFD NF, irradiated with laser at 405 nm and stained with propidium iodide (PI)24 h post irradiation. **a** Transmission (left and central columns) and PI fluorescence (right column) of cells treated with NPs_D2 loaded with HPPH and JB7-08. **b** NPs_D2 loaded with HPPH and JB17-08. Irradiated (laser scanned) area is marked by green dashed square.

## Data Availability

All data generated or analysed during this study are included in this published article and its additional information files.

## Abbreviations

^1^O_2_: singlet oxygen
NPs: nanoparticles
NIRFD: near infrared fluorescent dye
ABDA: 9, 10-anthracenediyl-bis(methylene)dimalonic acid
DLS: dynamic light scattering
DMEM: Dulbecco’s modified eagle’s medium
DMSO: dimethyl sulfoxide
FL: fluorescence
PBS: phosphate-buffered saline
PDI: polydispersity index
PDT: photodynamic therapy
PI: propidium iodide
PS: photosensitizer
ROS: reactive oxygen species
SWIR: short-wave infrared
NF: nanoformulation
NIR: near infrared
PAA: polyacrylamide
PNIPAM: poly-*N*-isopropylacrylamide
polySt: polystyrene
HPPH: 2-[1-hexyloxyethyl]-2-devinyl pyropheophorbide-alpha
AA: acrylamide
FI: FL imaging
