# Supplementary material for: Core–shell polymeric nanoparticles co-loaded with photosensitizer and organic dye for photodynamic therapy guided by fluorescence imaging in near and short-wave infrared spectral regions
Source: J Nanobiotechnology. 2020 Jan 23;18:19. doi: 10.1186/s12951-020-0572-1 (PMC6979398; doi:10.1186/s12951-020-0572-1)
Supplement: Supplementary file 1 — Additional file 1: Figure S1. Transmission electron microscopy (TEM) images of NPs_D2 with (a) and without (b) TEM contrast agent (phosphotungstic acid); NPs loaded PS and NIRFD without contrast agent (c). Figure S2. Fluorescence spectra of NF: polySt-poly(NIPAM-co-AA) NPs loaded with HPPH and dyes. a Fluorescence of HPPH emission loaded to NPs_D1 and NPs_D2 (excitation at 400 nm). b Fluorescence of NPs_D2 loaded with HPPH only and with HPPH and different concentration of JB 17-08; c Dependence of fluorescence intensity of JB 17-08 loaded NPs_D1 and NPs_D2 on dye concentration. Fluorescence was excited at 808 nm. Figure S3. Absorption (a) and fluorescence spectra (b, c) of HPPH and JB17-07 loaded to NPs_D2 before (black) and after (red) dialysis. Figure S4. Changes in absorption spectra of HPPH loaded to NPs_D2 under 532 nm laser irradiation. Samples were irradiated for 0,15,30 and 60 min. Figure S5. Confocal microscopy images of HeLa cells pre-incubated with HPPH/NIRFD NF, irradiated with laser at 405 nm and stained with propidium iodide (PI)24 h post irradiation. a Transmission (left and central columns) and PI fluorescence (right column) of cells treated with NPs_D2 loaded with HPPH and JB7-08. b NPs_D2 loaded with HPPH and JB17-08. Irradiated (laser scanned) area is marked by green dashed square. [file 12951_2020_572_MOESM1_ESM.docx]

Core-shell polymeric nanoparticles co-loaded with photosensitizer and organic dye for photodynamic therapy guided by fluorescence imaging in near and short-wave infrared spectral regions

O.M. Chepurna^1^, A. Yakovliev^1^, R. Ziniuk^1^, O. A. Nikolaeva^2^, S. M. Levchenko^1^, H. Xu^1^, M. Y. Losytskyy^2^, J. L. Bricks^3^, Yu. L. Slominskii^3^, L. O. Vretik^2*^, J. Qu^1*^, T. Y. Ohulchanskyy^1*^

^1^Key Laboratory of Optoelectronic Devices and Systems of Ministry of Education and Guangdong Province, College of Physics and Optoelectronic Engineering, Shenzhen University, Shenzhen, 518060, P. R. China

^2^ Taras Shevchenko National University of Kyiv, 01601 Kyiv, Ukraine

^3^Institute of Organic Chemistry, National Academy of Sciences of Ukraine, 02094 Kyiv, Ukraine

*Correspondence: [tyo@szu.edu.cn](mailto:tyo@szu.edu.cn), [l.vretik@univ.net.ua](mailto:l.vretik@univ.net.ua), [jlqu@szu.edu.cn](mailto:jlqu@szu.edu.cn)

**
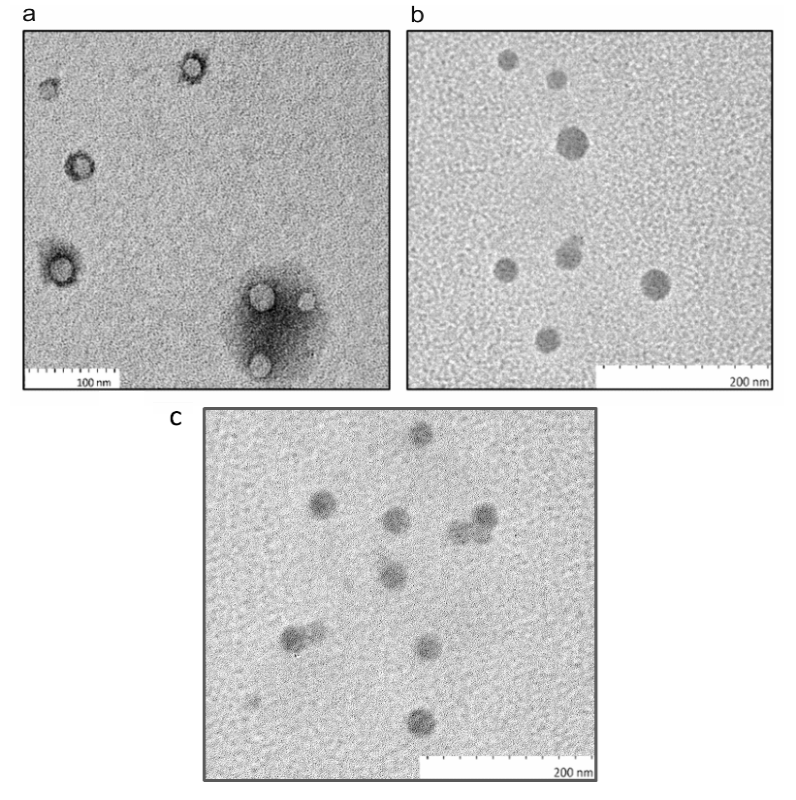
**

Figure S1 Transmission electron microscopy (TEM) images of NPs_D2 with (a) and without (b) TEM contrast agent (phosphotungstic acid); NPs loaded PS and NIRFD without contrast agent (c)


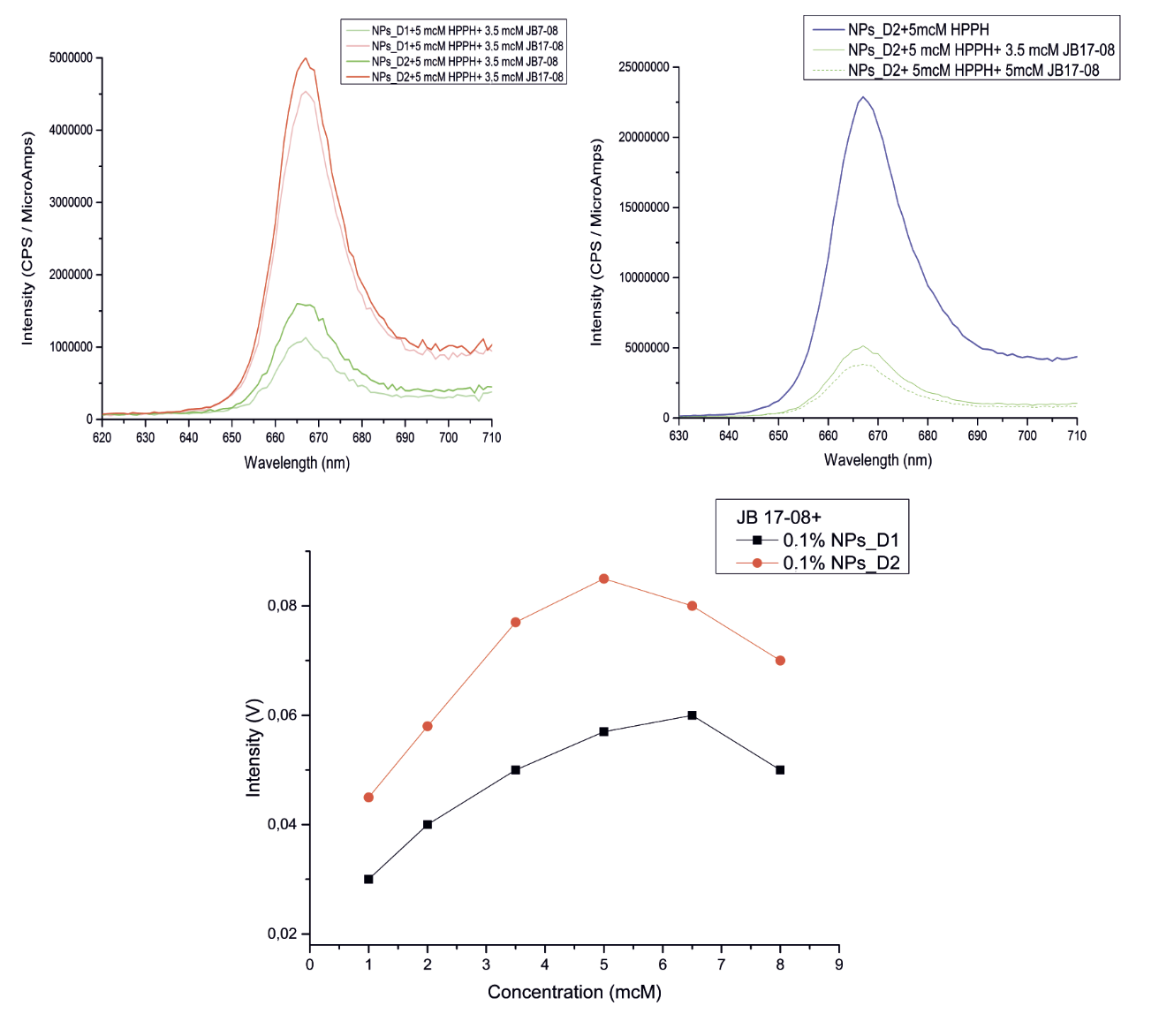


Figure S2

Fluorescence spectra of NF: polySt-poly(NIPAM-co-AA) NPs loaded with HPPH and dyes. **a** Fluorescence of HPPH emission loaded to NPs_D1 and NPs_D2 (excitation at 400 nm). **b** Fluorescence of NPs_D2 loaded with HPPH only and with HPPH and different concentration of JB 17-08; **c** Dependence of fluorescence intensity of JB 17-08 loaded NPs_D1 and NPs_D2 on dye concentration. Fluorescence was excited at 808 nm.

**

**

Figure S3

Absorption (a) and fluorescence spectra (b, c) of HPPH and JB17-07 loaded to NPs_D2 before (black) and after (red) dialysis


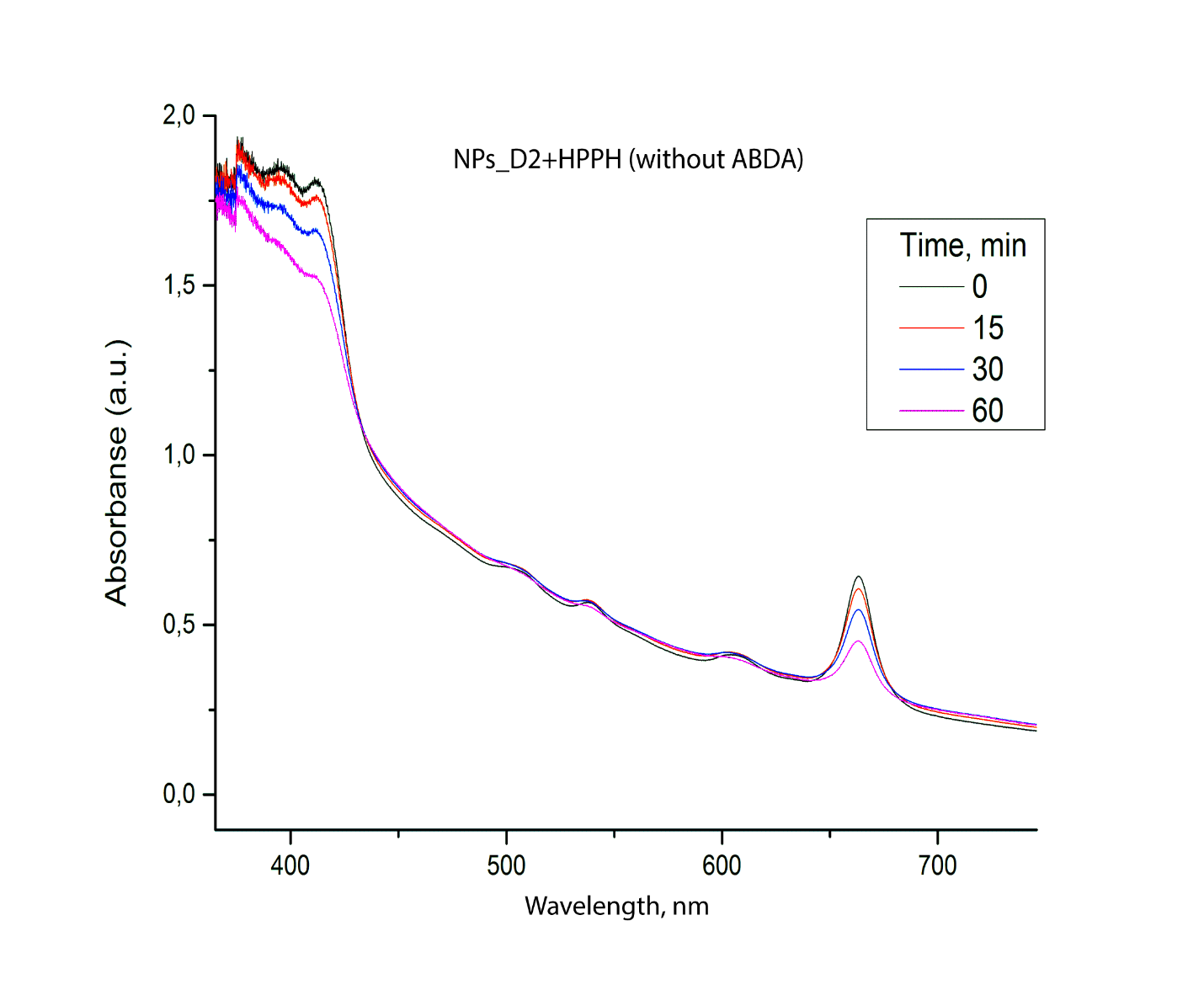


Figure S4

Changes in absorption spectra of HPPH loaded to NPs_D2 under 532 nm laser irradiation. Samples were irradiated for 0,15,30 and 60 min.


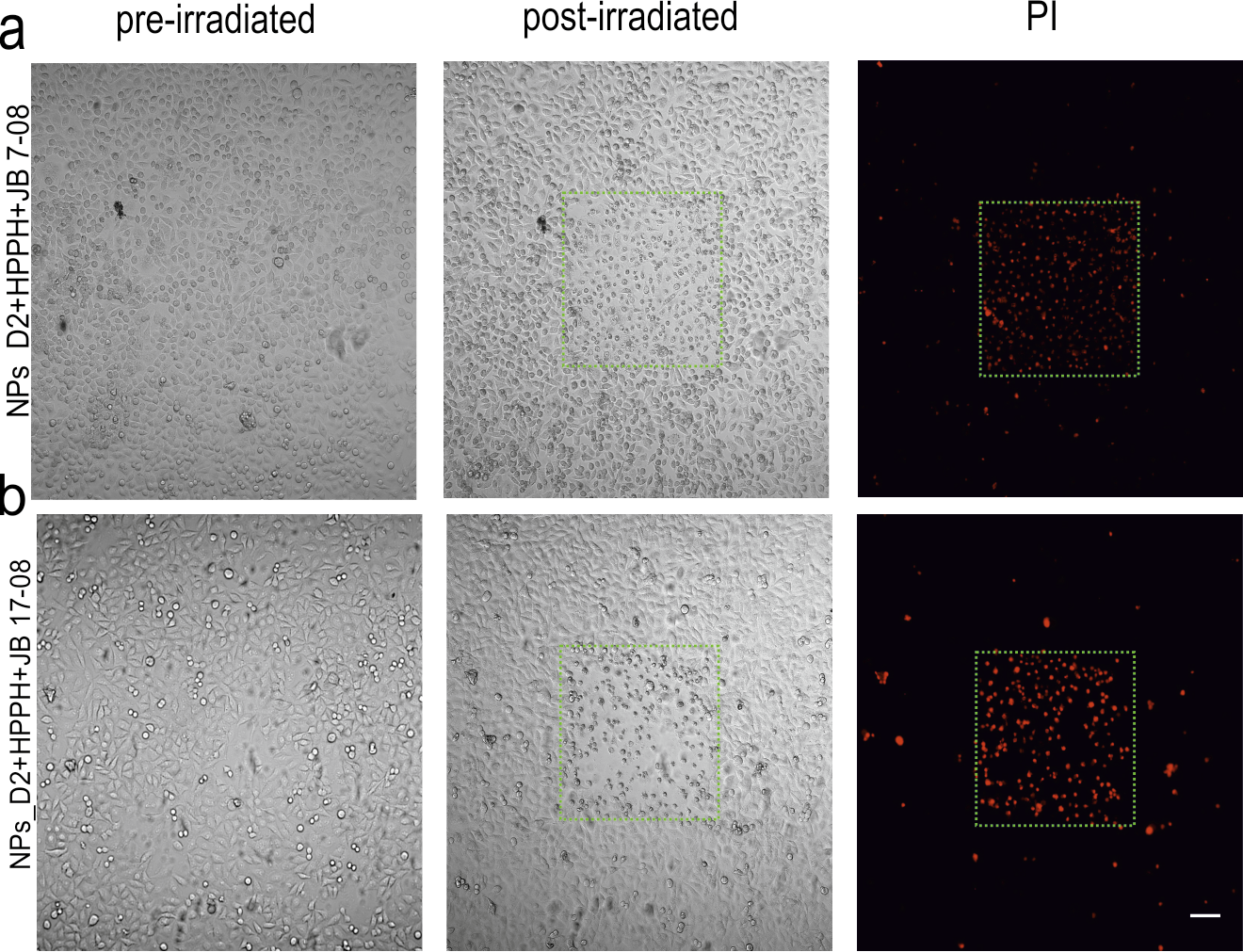


Figure S5

Confocal microscopy images of HeLa cells pre-incubated with HPPH/NIRFD NF, irradiated with laser at 405 nm and stained with propidium iodide (PI)24 h post irradiation. **a** Transmission (left and central columns) and PI fluorescence (right column) of cells treated with NPs_D2 loaded with HPPH and JB7-08. **b** NPs_D2 loaded with HPPH and JB17-08. Irradiated (laser scanned) area is marked by green dashed square.
